# Significant variations of dangerous exposures during COVID-19 pandemic in Italy: a possible association with the containment measures implemented to reduce the virus transmission

**DOI:** 10.1186/s12889-022-12860-3

**Published:** 2022-03-05

**Authors:** Felice Giordano, Valeria Margherita Petrolini, Domenico Spagnolo, Rosanna Maria Fidente, Lucrezia Lanciotti, Lucilla Baldassarri, Francesco Luca Moretti, Elena Brambilla, Davide Lonati, Azzurra Schicchi, Carlo Alessandro Locatelli, Rosa Draisci

**Affiliations:** 1 National Center for Chemicals, Cosmetics and Consumer Protection, Istituto Superiore di Sanità, Viale Regina Elena 299, 00100 Rome, Italy; 2 Toxicology Unit, Poison Centre and National Toxicology Information Centre, Istituti Clinici Scientifici Maugeri, IRCCS, Pavia, Italy

**Keywords:** Poison control centers, SARS-CoV-2 virus, Poisoning, Disinfectants, Hand sanitizers, Hazardous substances, Preventive health services, Public health surveillance

## Abstract

**Background:**

In response to the COVID-19 health emergency, mass media widely spread guidelines to stop the virus transmission, leading to an excessive and unaware use of detergents and disinfectants. In Italy and in other countries this tendency caused a significant increase of exposures to these products in 2020. Evaluating data collected by the Italian Pavia Poison Centre (PPC), this study intends to examine the relationship between the COVID-19 lockdown and the variations of exposures to specific product categories possibly associated to the containment measures implemented.

Simultaneously, this work shows the effectiveness of the European Product Categorisation System (EuPCS) in surveillance activities of dangerous chemicals.

**Methods:**

Exposure cases managed by the PPC during March–May 2020 (lockdown) and during the same months of 2017–2018-2019 were compared. Differences in categorical variables were tested with the Chi-square test. The level of significance was set at Alpha = .05. The study included all EuPCS groups but specifically focused on cleaners, detergents, biocides and cosmetics.

**Results:**

During the lockdown, calls from private citizens showed a highly significant increase (+ 11.5%, *p* < .001) and occupational exposures decreased (− 11.7%, *p* = .011). Among Cleaners, exposures to Bleaches slightly increased while Drain cleaning products went through a significant reduction (− 13.9%, *p* = .035). A highly significant increase of exposures to Disinfectants was observed (+ 7.7%, *p* = .007), particularly to those for surfaces (+ 6.8%, *p* = .039). Regarding Cosmetics, both handwashing soaps and gel products significantly increased (respectively: + 25.0, *p* = .016 and + 9.7%, *p* = .028).

Among children 1–5 years, the statistical significance is reached with exposures to Dishwashing detergents (+ 13.1%, *p* = .032), handwashing soaps (+ 28.6%, *p* = .014) and handwashing gel products (+ 16.8%, *p* = .010). Contrarily, Liquid Laundry Detergent Capsules decreased in a highly significant manner (− 25%; *p* = .001). The general severity of exposures showed a highly significant decrease (Moderate: − 10.1%, *p* = .0002).

**Conclusions:**

This study investigated the relationship between the COVID-19 lockdown and the variations of exposures to some product categories related to the containment measures. The results obtained support any action to be taken by Competent Authorities to implement measures for a safer use of cleaners/disinfectants. This paper shows the benefit in applying the EuPCS to categorize products according to their intended use, though an extension of this system to products not covered by CLP Regulation may be a further advantage.

## Background

Due to COVID-19 worldwide pandemic events, in the first months of 2020 there was a dramatic increase in the use of detergents/cleaners and disinfectants, both in health care and domestic settings. Starting on February 23rd 2020, the Italian government issued a series of guidelines intended to contain COVID-19 dissemination among the population [1]. The decree of March 4th 2020 specifically mentioned several actions, amongst all the frequent handwashing with hydro-alcoholic solutions and the disinfection of surfaces with chlorine or alcohol based products [2].

The National Decrees also used as sources the available information provided by CDC (Centers for Disease Control and Prevention), ECDC (European Centre for Disease Control and Prevention) and WHO (World Health Organization) about the use of substances active on viruses [3]. These warnings were advertised in the main mass media channels/newspapers/web, mixing both true and false information about use and application of detergents and disinfectants, leading to an increase in accidental exposures to these products [4, 5]. This finding is particularly relevant in domestic settings (i.e. use of not-appropriate products in relation to the intended target or field of application), while in professional ones a decrease was observed [4, 5]. On March–April 2020, Poison Centers (PCs) all over the world launched several alerts concerning the increasing trend of poisonings due to disinfectants. One of these alerts was spread by an Italian PC and mentioned the inhalation as the main route of exposure: children under 5 years were mainly exposed to disinfectants held in not-labelled bottles and left within reach of children, while adults underwent poisonings caused by the use of disinfectants to improperly treat face masks or by poisonous aerosols released by mixing different household products [6]. A first study based on data coming from the National Poison Data System of United States was published by Chang et al. [7], highlighting a sharp increase in alert calls for exposures to both cleaners and disinfectants at the beginning of March 2020. The study compared exposure information of the first quarter of 2020 with the same period of 2018 and 2019. Bleach-based products and non-alcohol and hand sanitizer products were the most common cause of such alerts. It was possible to observe an increase in the percentage of exposures through the inhalation route also in this case.

Subsequently, during 2020 and early 2021, many other scientific reports documented this occurrence [8, 9].

In Italy, the National Center for Chemicals, Cosmetics and Consumer Protection (CNSC-ISS) collects the exposure cases to dangerous chemical substances according to the EuPCS - European Product Categorization System [10, 11]. Referring to data coming from an Italian PC, the aim of this study was to evaluate the temporal association between the lockdown period due to COVID-19 pandemic and the variation of exposures to specific product categories (i.e. cleaning products, detergents, biocidal products and cosmetics) possibly associated to the containment measures implemented in Italy**.** This study also intends to show the effectiveness of applying the EuPCS categorization for surveillance activities carried out by PCs.

## Methods

### Data sources

In Italy, The Agreement of the Permanent Conference “Italian State- Regions”, 28th February 2008, defined rules and activities of PCs [12].

Currently, the Italian network involves ten PCs [13]. One of them is the Pavia Poison Centre (PPC) – National Toxicology Information Centre - a hospital based unit (24/7) where clinical toxicologists give precious advice and assist both physicians from the hospital emergency departments in diagnosing and managing poisonings and citizens requiring specialist consultations all over Italy. All consultations are recorded and significant cases are followed up to the outcome.

The occurrence of clinical cases due to non-pharmaceutical chemical agents is more than 25,000 per year (about the 40% of cases referred to all the Italian PCs) [14]. The PPC database records agents of exposure, demographic characteristics, route of exposure, reason for exposure, signs and symptoms, treatment and outcome variables.

According to the activity of toxicosurveillance for exposures to hazardous substances, the PPC transmits its data to CNSC-ISS.

### Study design

A retrospective analysis on PPC data was conducted. All cases of exposure to EuPCS product groups were included.

**Fig. 1:**
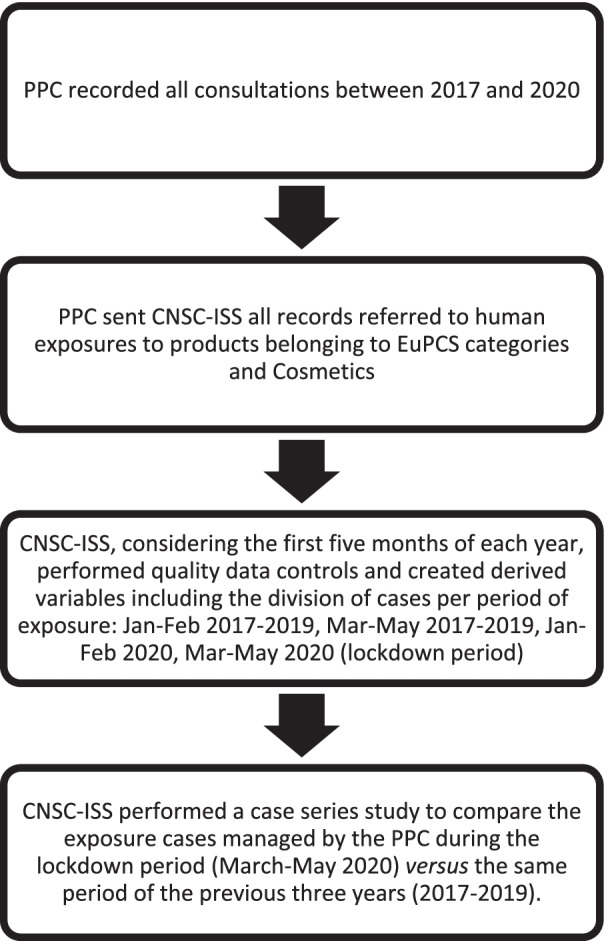
Main steps of the case series study performed

A case series study was designed to compare the exposure cases managed by the PPC during the lockdown period (pinpointed in March–May 2020 trimester) versus the same period of the previous 3 years (2017–2019). Percentages on the total of exposures managed in the first 5 months of each year were performed. In order to provide a better comprehension, the main steps of the case series study are summarized in Fig. 1.

### Variables

Descriptive analysis included patients’ demographic characteristics (gender and age), source of the call (hospital, non-hospital), exposure characteristics (intentional/ inadvertent), place of exposure, period_year (2017–2019 / 2020), period_month (January–February / March–May), route of exposure, symptoms, Poison Severity Score [15] and product categories involved. The latter variable is based on the EuPCS developed by ECHA (European Chemicals Agency). This system intends to facilitate the transmission of information on the intended use of a mixture for which a submission must be made according to Article 45 and Annex VIII of the Regulation (CE) 1272/2008 (CLP) and to support the statistical analysis of poisonings.

In Italy, products for hand hygiene not registered as disinfectants under the transitional period (i.e. containing active substances under revision according to the Reg (UE) 1062/2014 [16]) can be placed on the market as cosmetics. Being out of the scope of CLP Regulation, their categorization is based on the Regulation (CE) 1223/2009 [17].

To assign the correct product category some rules were fixed:

- Searching the web (also through the images) to obtain label information
- For biocidal product, confirm it by searching the trade name or the active substance in ECHA website (ECHA > Information on Chemicals > Biocidal Products)
- If technical information on the label was not available, the product category was assigned using the label claim.

### Case selection criteria

For the first 5 months of the years 2017, 2018, 2019 and 2020 all human exposure cases were extracted from the PPC database.

The analysis considered all the EuPCS third-level product categories.

Focusing on Cleaning, care and maintenance products (PC-CLN), Detergents and auxiliaries for laundry and dishwashing (PC-DET) and Biocidal products (PP-BIO), the following subcategories were specifically considered:

- PC-CLN-3: Bleaching products for cleaning or laundry use (excludes biocidal products)
- PC-CLN-5: Drain cleaning products
- PC-DET-1: Laundry detergents
- PC-DET-3: Dishwashing detergents
- PP-BIO-1: Biocidal products for human hygiene
- PP-BIO-2: Disinfectants and algaecides not intended for direct application to humans or animals.

The last two categories are included in the group of Disinfectants (Table 2), according to the Regulation (EU) No 528/2012 on biocides [18].

Among PC-DET-1 we also considered the subgroup of Liquid Laundry Detergent Capsules (LLDC). The analysis also concerned Cosmetics, which are not included in the EuPCS. Among them, the following subgroups were considered:

- Handwashing soaps (excludes biocidal products)
- Handwashing gel products (excludes biocidal products)

### Statistical analysis

Data were analysed using descriptive statistical methods. Differences in categorical variables were tested with the Chi-square test. The level of significance was set at Alpha = 0.05. For each EuPCS category, the observed cases (obs) and the expected ones (exp) in March–May 2020 were reported. Expected cases were calculated by applying the percentages of observed cases in March–May 2017–2020 on the total of cases occurred in the first 5 months of the same years. The percentage difference (% of increase) between observed and expected values was reported.

**Fig. 2:**
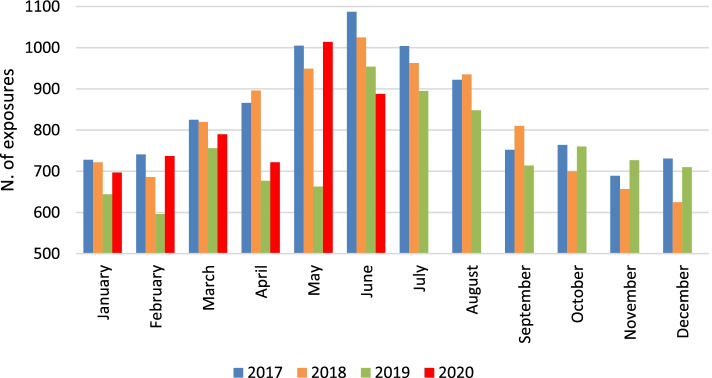
Distribution of monthly exposures managed by the PPC per year

Considering the total number of exposures selected in this study, a seasonality peak was observed in June in the years 2017–2018-2019 (Fig. 2). Exposures data of 2020 were available until June. In the first 3 months of 2020 and 2017–2019 the mean daily number of exposures reveals a similar trend (January: 22.5 in 2020 and 2017–2019; February: 25.4 in 2020 and 24.1 in 2017–2019, *p* = .240; March: 25.5 in 2020 and 25.8 in 2017–2019, *p* = .840). A significant lower mean daily number of exposures (24.1) was observed in April 2020 in comparison with April 2017–2019 (27.1), *p* = .025. On the contrary, in May 2020 a higher mean daily number of exposures was observed (May 2020: 32.7; May 2017–2019: 28.1; *p* = .005). During the lockdown period (March–May 2020) no statistically significant difference in the mean daily number of exposures compared to the same months of the previous years was observed (2020: 27.5 vs 2017–2019: 27.0; *p* = .641). Considering these evidences, the variations eventually observed during the lockdown period compared to the same months of 2017–2019 should not be affected by pre-existing trends or seasonal fluctuations.

When a statistically significant variation of the exposure frequency associated to the lockdown period exists, the effect size by Cohen *w* (omega) index was calculated. The statistical analysis was performed using IBM SPSS® Statistics Ver. 26 software.

## Results

**Fig. 3:**
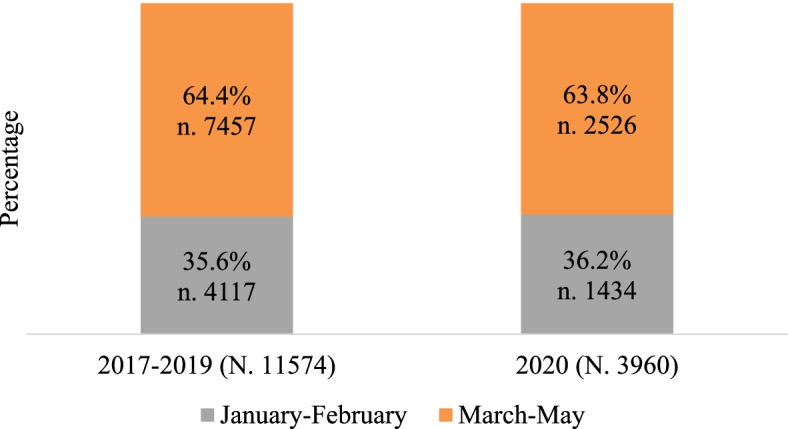
Exposure cases managed by the PPC: comparison between January–February and March–May of 2017–2019 and 2020

In applying the selecting criteria, 15,534 patients were identified by adding up the exposure cases of the first 5 months of 2017–2019 and 2020: 2017–2019 n. 11,574; 74.5% and 2020 n. 3960; 25.5% (Fig. 3). The distribution by months in 2020 [January–February: n. 1434 (36.2%); March–May: n. 2526 (63.8%)] does not show a statistically significant difference in comparison with 2017–2019 period [January–February: n. 4117 (35.6%); March–May: n. 7457 (64.4%)] (*p* = .467).

Table 1 shows the number of observed and expected biological and poisoning characteristics of patients exposed during the lockdown period (March–May 2020). Distribution by gender is comparable, even if there is a slight not significant decrease in observed male patients compared to the expected ones (− 2.5%; *p* = .077). Among age classes, a highly significant percentage difference highlights a reduction of exposures in patients aged 11–19 years (− 17.9%; *p* = .0003). The age class 1–5 years is the only one showing an increase in the lockdown period, although in a not statistically significant way (+ 2.8%; *p* = .099). The percentage difference among main circumstance categories is not statistically significant but the Occupational subcategory shows a highly significant decrease in comparison with the expected value (obs: n. 118; exp: n. 134; − 11.7%; *p* = .011). While the hospital calls show a highly significant decrease in March–May 2020 (− 10.8%; *p* < .001), the non-hospital ones undergo a highly significant increase (+ 10.5%; p < .001). This finding is mainly driven by private citizens (+ 11.5%; p < .001). The ocular route of exposure highlights a 6.8% increase in comparison with the expected frequency (obs: n. 166; exp: n. 156; *p* = .083), being statistically significant with the age class 1–5 years (obs: n. 72; exp: n. 64; + 12.5%; *p* = .039) (data not shown). During the lockdown period symptomatic cases showed a lowly significant decrease (− 2.7%; *p* = .047), but among those with PSS = Moderate the statistical significance of the decrease is high (− 10.1%; *p* = .0002).

**Table 1:** Biological and poisoning characteristics of patients exposed: observed and expected values in the lockdown period (March–May 2020)

Variables	March–May 2020 (Lockdown period)		% of increase	***p***-value (χ^**2**^)
	Observed N.	Expected N.		
Gender				
Male	1293	1325.9	−2.5	.077
Female	1233	1223.5	+ 0.8	.599
Unknown	–	0.5	−100	1
Age class (years)				
< 1	65	70.4	−7.7	.208
1–5	959	933.2	+ 2.8	.099
6–10	80	82.8	−3.4	.544
11–19	96	117.0	**−17.9**	**.0003**
20–65	1020	1027.3	−0.7	.659
66+	247	258.4	−4.4	.156
Unknown	59	54.8	+ 7.7	.291
Circumstance				
Inadvertent	2181	2187.5	−0.3	.786
Occupational	118	133.6	**−11.7**	**.011**
Intentional	328	339.6	−3.4	.242
Unknown	17	17.4	−2.3	.839
Caller				
Hospital	1279	1433.5	**−10.8**	**<.001**
Non-hospital	1247	1128.3	**+ 10.5**	**<.001**
Citizen	1083	971.1	**+ 11.5**	**<.001**
Route of exposure				
Ingestion	1701	1709.5	−0.5	.693
Inhalation	393	409.1	−3.9	.114
Dermal	197	208.5	−5.5	.103
Ocular	166	155.5	+6.8	.083
Injection	8	8.7	−8.0	*.745
Symptoms				
Yes	1413	1451.9	**−2.7**	**.047**
Minor	1102	1105.1	−0.3	.856
Moderate	288	320.4	**−10.1**	**.0002**
Severe-Fatal	23	27.0	−14.8	.139
Total	2526	2544.9	−0.7	.467

*Fisher Exact Test

Table 2 shows the observed and the expected number of commercial products, categorized by using the EuPCS, involved in inadvertent exposures occurred during the lockdown.

**Table 2:** Product categories (EuPCS) involved in patients’ inadvertent exposures: observed and expected values in the lockdown period (March–May 2020)

Variables	March–May 2020 (Lockdown period)		Increase (%)	***p***-value (χ^**2**^)
	Observed N.	Expected N.		
Adhesives and sealants (PC-ADH)	26	27.5	−5.5	.557
Air care products (PC-AIR)	35	36.4	−3.8	.698
Products for animals (PC-ANI)	2	1.5	+ 33.3	*1.000
Art materials (PC-ART)	44	39.7	+ 10.8	.244
Cleaning, care and maintenance products (excludes biocidal products) (PC-CLN)	840	848.3	−1.0	.582
Bleaching products for cleaning or laundry use (PC-CLN-3)	322	308.8	+ 4.3	.132
Drain cleaning products (PC-CLN-5)	59	68.5	**−13.9**	**.035**
Colourants (PC-COL)	1	1.4	−28.6	*1.000
Construction products (PC-CON)	10	8.5	+ 17.6	*.465
Detergents and auxiliaries for laundry and dishwashing (excludes biocidal products) (PC-DET)	278	281.1	−1.1	.720
Laundry detergents (PC-DET-1)	66	76.7	**−14.0**	**.024**
- LLDC	35	46.1	**−24.1**	**.002**
Dishwashing detergents (PC-DET-3)	127	119.1	+6.6	.138
E-liquids and mixtures for electronic cigarettes (PC-ELQ)	13	14.0	−7.1	.632
Fertilisers and fertilising products (PC-FER)	45	46.4	−3.0	.653
Fuels (and fuel additives) (PC-FUE)	70	78.6	−10.9	.081
Inks, toners and related printing materials (PC-INK)	12	16.8	**−28.6**	**.031**
Paints and coatings (and related auxiliaries) (PC-PNT)	36	37.4	−3.7	.683
Pyrotechnic products (PC-PYR)	–	–	–	–
Products for chemical or technical processes (PC-TEC)	121	124.2	−2.6	.618
Biocidal products (PP-BIO)	382	364.3	**+ 4.9**	**.032**
-Disinfectants (PP-BIO-1 to PP-BIO-5)	265	246.0	**+ 7.7**	**.007**
Biocidal products for human hygiene (PP-BIO-1)	79	72.6	+ 8.8	.108
Disinfectants and algaecides not intended for direct application to humans or animals (PP-BIO-2)	182	170.4	**+6.8**	**.039**
Plant protection products (excludes biocidal products) (PP-PRD)	76	80.7	−5.8	.161
Cosmetics	289	276.5	+ 4.5	.145
Handwashing soaps (excludes biocidal products)	23	18.4	**+ 25.0**	**.016**
Handwashing gel products (excludes biocidal products)	52	47.4	**+ 9.7**	**.028**

*Fisher Exact Test

The percentage difference of Art materials undergoes an unexpected 10.8% increase, notwithstanding the lack of statistical significance (*p* = .244). Among Cleaning**,** care and maintenance products, the number of observed Bleaching products (PC-CLN-3) is higher than the expected one (obs: 322; exp: 309; + 4.3%; *p* = .132). This result becomes highly statistically significant among male patients aged 20–65 years (obs: 56; exp: 47; + 19.1%; *p* = .008) (data not shown). On the contrary, a statistically significant decrease is observed for Drain cleaning products (− 13.9%; *p* = .035). Here too, this difference becomes greater among male subjects belonging to the age class 20–65 years (obs: 20; exp: 30; − 33.3%; *p* = .0002) (data not shown). In March–May 2020 Laundry detergents prove to be less involved in inadvertent exposures than expected (obs: 66; exp: 77; − 14.0%; *p* = .024). This finding seems to be associated with the decrease of LLDC (obs: 35; exp: 46; − 24.1%; *p* = .002), mainly driven by the age class 1–5 years (obs: 33; exp: 44; − 25%; *p* = .001) (data not shown). In contrast with other PC-DET subcategories, Dishwashing detergents highlight a 6.6% increase compared to the expected values (*p* = .138), becoming statistically significant in the age class 1–5 years (obs: 70; exp: 62; + 13.1%; *p* = .032) (data not shown). For products belonging to the Inks, toners and related printing materials (PC-INK), there is a statistically significant decrease (*p* = .031) in observed exposures (n. 12) compared to the expected value (n. 17). An increased number of Biocidal products is observed (obs: 382; exp: 364; + 4.9%; p = .032), probably due to Disinfectants (obs: 265; exp: 246; + 7.7%; *p* = .007) and in particular to Disinfectants and algaecides not intended for direct application to humans or animals (obs: 182; exp: 170; + 6.8%; *p* = .039). During the lockdown period, the proportion of Cosmetics used for personal hygiene increased too: Handwashing soaps showed a 25.0% increase (*p* = .016) while Handwashing gel products registered a 9.7% increase (*p* = .028). A high statistical significance is also reached with children aged 1–5 years (Handwashing soaps: obs: 18; exp: 14; + 28.6%, *p* = .014; Handwashing gel products: obs: 32; exp: 27; + 16.8%, *p* = .010) (data not shown). Generally, Cohen’s *w* (omega) index, calculated when statistically significant effects were found, remains below 0.20. The highest magnitude of effect was found for Handwashing gel products (Cohen’s *w* = 0.22, almost medium effect size) and for Handwashing soaps (Cohen’s *w* = 0.30, medium effect size).

## Discussion

This research based on PC data aimed at verifying whether during COVID-19 pandemic lockdown there were variations of exposure frequencies to specific product categories possibly associated to the containment measures and to the recommendations disclosed by the health authorities.

Italy has been the first Western country to adopt the most restrictive lockdown measures in the first months of 2020. Since PPC provides consultancies for the whole national territory, covering about 40% of those referring to all ten Italian PCs [13], the results obtained are representative of the entire Italian situation.

This is the first study using all EuPCS third-level categories and some of their sub-categories. Among all EuPCS categories, the attention was specifically focused on those involved in disinfecting and cleaning activities.

Considering the biological characteristics, the observed number of male patients slightly decreased during the lockdown period (March–May 2020) in comparison with the expected value. This finding may be attributed to women taking greater responsibilities to manage the disinfection of different settings, particularly the domestic ones. On the other hand, exposures in workplaces decreased in a highly significant way. This evidence is in line with the reduction of occupational injuries during COVID-19 lockdown reported by the National Institute for Insurance against Accidents at Work (INAIL) [19]. Soave et al. [5] showed a 200% increase of occupational exposures during January–May 2020 but the small population involved does not allow making a comparison with the present study.

Among age groups, the class 1–5 years shows a not statistically significant increase during the lockdown period. Children’s exposures to disinfectants are a matter of great concern for Li et al. [20] (2020), who highlight that, due to their more frequent mouthing activities, children have consistent higher exposures to disinfectants than other age classes. The percentage difference calculated for the age class 11–19 years shows a highly statistically significant reduction of exposures during the lockdown. In fact, it seems that young people have significantly changed their lifestyle habits in this period, spending more time at home and mainly preferring sedentary activities [21]. These unhealthy behaviours might have avoided other physical activities involving accidental exposures to dangerous chemicals. On the other hand, the reduction of physical activities may have led to a decrease in mental wellbeing [22].

While normally PPC mainly receives hospital calls (about 82% of consultancies come from emergency departments), a highly significant decrease was observed during the lockdown period. On the other hand, the number of non-hospital calls significantly increased, particularly because of calls from private citizens. This evidence could highlight a tendency of the population not to access hospitals or general practitioners if not strictly necessary, a fact in line with the recommendations given during COVID-19 pandemic. Other Italian [5, 23] and European [4] PCs also reported this trend.

The ocular route of exposure shows an increased percentage in comparison with the expected frequency, reaching the statistical significance with children aged 1–5 years. A statistically significant increase of paediatric ocular exposures to alcohol-based hand sanitizers (ABHS) from April to August 2020 are reported by Martin et al. [24], though considering subjects younger than 18 years.

In March–May 2020 symptomatic cases decreased in a lowly significant way, but among those with a Moderate PSS the statistical significance is high. This finding is mainly driven by a decrease of occupational exposures; in fact, high severities are significantly linked to this kind of exposures (*p* = .0002) (data not shown). Also Le Roux et al. [4] detected a significant decrease of exposures at work (− 45.5%; *p* < .001) in March–April 2020 in comparison with the same period of the previous 2 years, and at the same time they observed a decrease in severity (moderate/severe − 17.2%). On the contrary, data from US PCs, where containment measures were less restrictive in that period, did not report the same decrease [8].

Moreover, fewer intentional exposures and the increased percentage of 1–5 years’ children’s exposures may have also contributed to a lower severity of the clinical picture: in many cases the effectiveness of children’s exposures is uncertain so most of them have little clinical relevance.

To categorize the products included in this study, the EuPCS developed by ECHA was used [11]. The advantage of this tool is that the intended use is easier to assign in comparison with other categorizations based on chemical structures. Nevertheless, ambiguous categorizations are still possible. Another limitation, in considering the purpose of a PC, is that this categorization system covers only product categories needing CLP classification, excluding other potentially toxic agents like cosmetics, animals, plants, food, drugs, tobacco (only E-liquid for E-cig is mentioned in the EuPCS). A certain advantage is that some product categories driven by specific regulations (Regulation (EU) No 528/2012 for Biocidal products, Regulation (EC) No 1107/2009 for Plant protection products [25]) maintain their original categorization in the EuPCS.

In this research, EuPCS allowed the categorization of products not only for consumer use, but also for professional and industrial use.

Among Cleaning, care and maintenance products (PC-CLN), Bleaching products for cleaning or laundry use (PC-CLN-3) and Drain cleaning products (PC-CLN-5) are the subcategories of most interest. Regarding the first, a 4.3% increase in exposures during the lockdown period was detected and the difference becomes highly statistically significant with male subjects belonging to the adult class 20–65 years. Since this evidence cannot be observed neither among female subjects nor among other age classes, it could be related to mistakes in handling these products made by non-professional and non-skilled/habitual users. Other authors commented the increase in household product exposures observed in many developed countries arguing that the probable reason for the high incidence detected could be ascribed to careless storage, ignorance, non-compliance with prescribed instructions for use and negligible parental supervision in case of children [26]. In the European Union a survey highlighted that the 70% of European citizens use the label to obtain information about the potential danger of a chemical, although chemical hazard pictograms are usually not very understood [27]. A more recent U.S. survey verified an important knowledge gap in the safe use of cleaners and disinfectants among the adult population [28]. Inadvertent exposures indicated that the general public/non-professionals pay little or no attention to the instructions for use of cleaners and disinfectant products. Also Chang et al. [7], in evaluating data from American PCs, found that, among cleaner categories, bleaches accounted for the largest percentage of the increase (n. 1949; 62.1%). Increased exposures to bleaches seem to be temporally subsequent to advises reported in all the main Italian mass media including guidelines issued to prevent COVID-19 circulation, recommending to disinfect surfaces with chlorine and alcohol based products. Fortunately, bleach intoxication from inhalation or ingestion of small amounts due to accidental reasons is not normally of clinical significance [29]. This evidence may further explain the low percentage of moderate/severe cases registered in March–May 2020. Considering the subgroup of Drain cleaning products (PC-CLN-5), a significant decrease in comparison with the expected value was observed. Moreover, among gender and age classes, the statistically significant difference becomes greater with male subjects of the adult class 20–65 years. Since these products are often referred to work contexts, this finding proves to be in line with the decrease of occupational exposures.

Among Detergents and auxiliaries for laundry and dishwashing (excludes biocidal products) (PC-DET), two different trends could be observed: on the one hand, there was a highly statistically significant decrease of Laundry detergents (PC-DET-1) probably caused by LLDC exposures of children aged 1–5 years; on the other, a significant increase in exposures of the same age class was detected for Dishwashing detergents (PC-DET-3). Differently, during COVID-19 crisis, Gulamhusein and Sabri [30] saw an increase in ocular exposures to LLDC among Canadian children. When investigating American NDPS data among children aged < 6 years, Gaw et al. [31] saw a declined trend by 55.5% from 2015 to 2017.

A possible explanation to the opposite tendencies observed could be as follows: with regard to Laundry detergents, the confinement reduced the possibility to go out and so the frequent need to wash clothes. On the other side, confinement doubtlessly led to an increase in the number of meals consumed at home, with a consequent increase in dishwashing activities. This high percentage in the age class 1–5 years underlines the need of a greater parental supervision when using products not suitable for children.

Regarding Biocidal products, a statistically significant increase during the lockdown period was observed. This result is probably due to Disinfectants. Chang et al. [7] observed a similar trend, although the statistical significance was not evaluated. Increased calls regarding household disinfectants were registered by several PCs around the world [5, 8, 9, 32]. In the present study, the statistical significance was specifically reached for Disinfectants and algaecides not intended for direct application to humans or animals, while Biocidal products for human hygiene (PP-BIO-1) only show a not statistically significant increased percentage. In the recent past, Wieck et al. [33] performed a study to investigate the knowledge about the appropriate use of biocidal products through a standardised questionnaire fed to German consumers. The research highlighted how only the 21% of consumers provided the exact definition of biocide. Nevertheless, the majority of the interviewees seemed not to be aware when and where they were actually using specific biocidal products. As a matter of fact, the product types mentioned in the interview often did not correspond to those the researchers actually found in interviewees’ houses. The highest percentage of the interviewees failed to mention disinfectants used for surfaces (17%). This type of biocides (PP-BIO-2) are often perceived as simply cleaners with a lower risk associated compared, for example, to Insecticides (PP-BIO-18). A survey of the Joint Research Centre of the European Commission [34] highlighted that more than a half of EU citizens always read the instructions before using pesticides and insecticides, but the reliance on the instructions is less common for other categories of chemical products. This underestimation of the risk could have led to dangerous exposures especially regarding disinfectants for surfaces frequently used in COVID-19 period.

Considering Cosmetics, both Handwashing soaps and Handwashing gel products show a statistically significant increase in March–May 2020 with respect to the expected value. In both cases, children aged 1–5 years mainly drive this increase**.** This evidence is consistent with the findings of Hakimi and Armstrong [35], who pointed out a significant increase in daily calls to US PCs regarding paediatric hand sanitizer exposures in March 2020 compared to March 2018–2019 and to January–February 2020, yet referring to children aged 12 years and younger.

### Limitation to the study

As mentioned before, ambiguous categorizations using the EuPCS are possible. Furthermore, the trade name of the products is sometimes not completely reported so it was not easy to identify the right EuPCS third-level category. For certain products, the statistical significance highlighted an association between the increasing frequency of exposures and the lockdown period, but the magnitude of the effect (Cohen’s *w* index) is generally small.

## Conclusions

The present study highlighted the variations of dangerous exposures to specific product categories during COVID-19 lockdown, by investigating the possible connection with the containment measures implemented in Italy. Both increase and decrease of exposures to certain product categories (i.e. cleaning products, detergents, biocidal products and cosmetics) were observed.

These findings are meant to be an encouragement for the Competent Authorities involved in public health matters to implement preventative measures for the general population, with awareness raising campaigns about the safe use of cleaners and disinfectants, as Italy carried out through the National Institute for Health website [36]. Even though already present in well prepared labels, further visibility should be provided to fundamental, although basic, warnings, such as: read the label carefully, use the indicated dilution of the product, do not mix different products if not specifically mentioned in the label, use products in well-ventilated areas, keep out of the reach of children.

This work is also an interesting example of the practical application of the EuPCS in an epidemiological study, used to categorize commercial products causing exposures/intoxications. EuPCS proved to be very useful when categorizing products according to the intended use is necessary. Nevertheless, an extension of the EuPCS to include those categories not covered by CLP Regulation may represent a further advantage for PCs.

## Data Availability

The datasets used and analysed during the current study are available from the corresponding author on reasonable request.

## Abbreviations

PPC: Pavia Poison Centre
EuPCS: European Product Categorisation System
CDC: Centers for Disease Control and Prevention
ECDC: European Centre for Disease Control and Prevention
WHO: World Health Organization
PC(s): Poison Center(s)
CNSC-ISS: National Center for Chemicals, Cosmetics and Consumer Protection-Istituto Superiore di Sanità
ECHA: European Chemicals Agency
PC-CLN: Cleaning, care and maintenance products
PC-DET: Detergents and auxiliaries for laundry and dishwashing
PP-BIO: Biocidal Products
PC-CLN-3: Bleaching products for cleaning or laundry use
PC-CLN-5: Drain cleaning products
PC-DET-1: Laundry detergents
PC-DET-3: Dishwashing detergents
PP-BIO-1: Biocidal products for human hygiene
PP-BIO-2: Disinfectants and algaecides not intended for direct application to humans
PC-INK: Inks, toners and related printing materials
LLDC: Liquid Laundry Detergent Capsules
ABHS: Alcohol-based hand sanitizers
PP-BIO-18: Insecticides, acaricides and products to control other arthropodes
